# Surveillance for Unexplained Deaths and Critical Illnesses

**DOI:** 10.3201/eid0802.010165

**Published:** 2002-02

**Authors:** Rana A. Hajjeh, David Relman, Paul R. Cieslak, Andre N. Sofair, Douglas Passaro, Jennifer Flood, James Johnson, Jill K. Hacker, Wun-Ju Shieh, R. Michael Hendry, Simo Nikkari, Stephen Ladd-Wilson, James Hadler, Jean Rainbow, Jordan W. Tappero, Christopher W. Woods, Laura Conn, Sarah Reagan, Sherif Zaki, Bradley A. Perkins, for the Unexplained Deaths, Critical Illness Working Group

**Affiliations:** * Centers for Disease Control and Prevention, Atlanta, Georgia, USA; † Stanford University, Stanford, California, USA; ‡ the Emerging Infections Program, Portland, Oregon, USA; § the Emerging Infections Program, Hartford, Connecticut, USA; ¶ the Emerging Infections Program, San Francisco, California, USA; # the Emerging Infections Program, Minneapolis, Minnesota, USA

**Keywords:** emerging infectious diseases, unexplained infectious diseases, 16S polymerase chain reaction

## Abstract

Population-based surveillance for unexplained death and critical illness possibly due to infectious causes (UNEX) was conducted in four U.S. Emerging Infections Program sites (population 7.7 million) from May 1, 1995, to December 31, 1998, to define the incidence, epidemiologic features, and etiology of this syndrome. A case was defined as death or critical illness in a hospitalized, previously healthy person, 1 to 49 years of age, with infection hallmarks but no cause identified after routine testing. A total of 137 cases were identified (incidence rate 0.5 per 100,000 per year). Patients’ median age was 20 years, 72 (53%) were female, 112 (82%) were white, and 41 (30%) died. The most common clinical presentations were neurologic (29%), respiratory (27%), and cardiac (21%). Infectious causes were identified for 34 cases (28% of the 122 cases with clinical specimens); 23 (68%) were diagnosed by reference serologic tests, and 11 (32%) by polymerase chain reaction-based methods. The UNEX network model would improve U.S. diagnostic capacities and preparedness for emerging infections.

The 1992 Institute of Medicine report--Emerging Infections, Microbial Threats to Health in the United States (*1*)--highlighted the need for a more effective means to detect emerging infectious diseases. In response to this report and as part of the Emerging Infections Program (EIP) (*2*), the Centers for Disease Control and Prevention (CDC) collaborated with state health departments and academic institutions to develop a pilot surveillance strategy for early detection of new and unrecognized infectious diseases in the United States. This project--Surveillance for Unexplained Deaths and Critical Illnesses Due to Possibly Infectious Causes--was developed on the basis of two observations. The first was the realization that supposedly new infectious diseases identified in the United States in recent decades had been occurring long before they were recognized and identified. The second was important progress in molecular diagnostic methods, which in some instances has allowed new infectious agents to be identified and characterized with molecular probes, making in vitro cultivation unnecessary.

In 1995, we initiated population-based surveillance for unexplained deaths and critical illnesses due to possibly infectious etiologies (UNEX) at four U.S. sites. The objectives of this effort were to define the incidence, epidemiologic features, and possible causes of these deaths and illnesses; create a bank of clinical specimens for future testing as new pathogens and methods are identified; and assist in building U.S. capacity for detecting and responding to uncommon and previously unrecognized pathogens. This report describes the methods we developed to reach these goals and the results of the first 3.5 years of surveillance.

## Methods

### Surveillance Sites

Population-based surveillance for UNEX was initiated on May 1, 1995, among persons 1 to 49 years of age residing in the San Francisco Bay area (Alameda, Contra Costa, and San Francisco Counties) of California (n=2,168,810); in New Haven County, Connecticut (n=556,592); in the entire state of Minnesota (n=3,419,760); and among persons 1 to 39 years of age residing in Oregon (n=1,544,466).^2^All these sites were participants in the EIP, and the total population targeted for surveillance was 7.7 million. We report the results of surveillance for cumulative cases through December 31, 1998.

### Case Definition

An UNEX case was defined as illness in a previously healthy resident of a surveillance area who was 1 to 49 years old (1 to 39 years old in Oregon) and who died or was hospitalized with a life-threatening illness with hallmarks of an infectious disease for which no cause was identified through routine testing initiated by health-care providers. A previously healthy person was defined as a patient without a preexisting known systemic, chronic medical illness diagnosed before the acute onset of the UNEX. Such preexisting conditions included malignancy; HIV infection; chronic cardiac, pulmonary, renal, hepatic, or rheumatologic disease; or diabetes mellitus. Patients were also excluded from the study if they had received any immunosuppressive therapy, had evidence of toxic ingestion or exposure, had trauma before their illness, or acquired their illness ≥48 hours after hospital admission (Appendix I).

A life-threatening illness was defined as any illness requiring admission to an intensive-care unit (ICU). Hallmarks of an infectious disease were defined as the following: fever or history of fever, leukocytosis, histopathologic evidence of an acute infectious process, or a physician-diagnosed syndrome consistent with an infectious etiology, including encephalitis or meningitis, fulminant hepatitis or hepatic failure, myocarditis, adult respiratory distress syndrome, respiratory failure, or sepsis.

### Case Finding and Ascertainment

Patients meeting the case definition were sought at surveillance sites through various mechanisms. Practicing clinicians in all surveillance sites were informed about the project through letters and bulletins and presentations at local and regional professional society meetings. Personnel at some surveillance sites attempted to identify cases more actively through regular communications with persons working in ICUs and local medical examiners or through routine review of ICU admission records. Physicians and other health professionals were asked to report suspected cases by telephone to local surveillance site personnel. When a case was reported, a screening form was completed to determine if the patient met the case definition. This surveillance system was not designed to provide timely reporting or testing.

## Surveillance Audit

To evaluate the sensitivity of the surveillance system, personnel at all surveillance sites conducted a retrospective review of death records from their surveillance areas, and three sites (California, Connecticut, and Oregon) reviewed all hospital discharge data in their areas for a period of at least 1 year. All death certificates for the age groups included in the surveillance were reviewed for the presence of specific International Classification of Disease codes (ICD-9), selected for their potential to identify unexplained deaths due to possibly infectious causes (*3*). Persons whose death records included ICD-9 codes indicating a disqualifying underlying medical condition were excluded. Once potential cases were identified, the patients’ medical records were reviewed. If the records were not available, the primary physician was contacted to determine if the patient met the surveillance case definition. The sensitivity of the surveillance system for detecting deaths (S_D_) was calculated by dividing the number of deaths (D1) detected through surveillance alone by the total number of deaths (D1+D2) detected through both surveillance and death record review (D2): S_D_=D1/D1+D2. The sensitivity of the surveillance system (S_C_) for detecting critical illness cases was calculated by dividing the number of such cases (C1) detected through surveillance alone by the total number of cases (C1+C2) found through both surveillance and hospital discharge review (C2): S=C1/C1+C2.

## Collection of Clinical Information and Specimens

For patients meeting the case definition, surveillance site personnel completed a case report form that included demographic, epidemiologic, and clinical information. This information was collected through interview of physicians caring for the patient, review of the medical record, and contact with the patient or the patient’s family. Cases were assigned a clinical syndrome depending on the predominant system involved, on the basis of information provided by the physician. These syndromes included neurologic (encephalitis, meningitis), cardiac (myocarditis, pericarditis, endocarditis), respiratory (pneumonitis), and hepatic (hepatitis). Syndromes such as sepsis, in which no predominant organ system was involved, were classified as “other.” The hospital laboratories were requested to save all remaining clinical specimens obtained as part of routine clinical management, including biopsies and autopsies.

## Laboratory Testing

For the first 2 years of the study, the project investigators selected diagnostic tests individually for each case. Decisions were made on the basis of clinical, epidemiologic, and histologic data; previous laboratory testing ordered by the health-care providers; and availability, timing, quality, and quantity of clinical specimens. In the third year of the project, based on information gained to date, a set of standardized syndrome-specific laboratory testing protocols was developed for respiratory, neurologic, cardiac, and hepatic syndromes (Appendix II).These protocols prioritized testing based on available clinical and epidemiologic information and a differential diagnosis; they guided a first round of laboratory testing which, if negative, prompted a customized second round of testing. Cases that did not fit any of these four syndromes were discussed by the project investigators on an individual basis.

### Histopathologic Testing

Whenever possible, in addition to initial examination by local pathologists, tissue specimens were examined by CDC pathologists to help guide further laboratory testing decisions. CDC pathologists have available a unique set of antibodies and probes for immunohistochemistry (IHC) and in-situ hybridization (ISH);^3^ these and other special studies, such as chemical stains, were selected based on all available case information. IHC tests were performed by a two-step indirect immunoalkaline phosphatase technique with various antibodies (*4*). ISH tests used digoxigenin-labeled probes with an immunoalkaline phosphatase staining protocol (*5*). Positive and negative controls were run in parallel with case specimens.

### Testing for Viral Pathogens

The California Department of Health Services (CDHS) Viral and Rickettsial Diseases Laboratory was the primary testing site for viral pathogens other than the hepatitis viruses. Serologic tests were available for immunoglobulin (Ig) G directed against 19 viral pathogens and for IgM directed against 14 of these.^4^ When only a single serum specimen was available, the presence of both IgM and IgG was assessed, either by enzyme immunosorbent assay (EIA), indirect immunofluorescence assay (IFA), or both (*6*). Paired sera were tested by EIA or IFA for increase in IgG titer. Additional testing included nucleic acid amplification by polymerase chain reaction (PCR) for selected viral pathogens if adequate specimens were available (*7*–*9*). An increase in IgG titer by EIA was interpreted as evidence of recent or current infection if the ratio of convalescent- to acute-phase indices was ≥1.5; an index is determined by the equation (optical density [OD]-positive antigen - OD-negative antigen)/predetermined positive threshold OD (usually 0.1). The CDHS diagnostic assays for IgM to B19V, *Cytomegalovirus*, *Hantavirus* (SNV), herpes simplex virus, MeV, MuV, RUBV, SLEV, VZV, and WEEV have varying minimum positive values, with indices from 1.0 and 2.0. For the enterovirus IgM assay, which detects the presence of enterovirus group antibody in serum, a ratio of OD-positive antigen to OD-negative antigen ≥2.26 was considered positive. Agents tested by IFA were considered positive if a fourfold or greater rise in titer was detected. IFA IgM assays were considered positive if the staining pattern was distinct for that agent at the appropriate serum dilution.

### Bacterial Broad-Range Ribosomal DNA (rDNA) PCR

DNA extraction from clinical specimens was performed as described (*10*,*11*). All clinical specimens tested with the broad-range bacterial rDNA PCR were analyzed by using at least one of the three following primer pairs: fD1mod (positions 8-27 in *Escherichia coli* 16S rRNA gene) (*12*) and 16S1RR-B (575-556) (*13*); 8F2 (8-27) and 806R (806-787); and 515F (515-533) and 13R (1390-1371). PCR products were characterized by direct sequencing or cloning and sequencing, followed by comparison with rDNA sequences available in GenBank (*11*).

## Criteria for Causation

Cases were defined as having definite, probable, possible, or no microbial etiology (Table 1). These levels of certainty for the causal role of an infectious agent reflected the integration of several factors, including the relationship of anatomic site of detection to site of disease, reliability of the method, and whether the putative agent was a known cause of the clinical syndrome under investigation. Cases were classified as explained if results showed a definite or probable disease cause and as unexplained if results indicated a possible infectious cause or none at all.

**Table 1:** Classification of laboratory test results and cases,^a^ surveillance for unexplained death and critical illness possibly due to infectious causes (UNEX), 1995-1998

A	B	C
1. Detection of organism by culture from involved site^b^	1. Detection of organism by culture, IF, IHC, IEM, ISH, or PCR^c^ in blood or clinically relevant site^d^	1. Detection of organism by culture, IF, IHC, IEM, ISH, or PCR from uninvolved, but nonmucosal, noncutaneous site
2. Detection of organism by direct immunologic staining (i.e., IF, IHC,IEM) at involved site	2. Positive serology: ≥4-fold change in IgG/IgA titer or significantly elevated IgM titer	
3. Detection of organism by DNA/RNA ISH at involved site	3. Detection of organism by EM^e^ at involved site	
4. Detection of organism by PCR^f^ at involved site	4. Detection of other specific microbial antigen at characteristic site (e.g., urine, CSF)	

^a^Case classification: A case was considered to have a definite explanation if the organism was a well-recognized cause of syndrome and there was one test from column A or 2 from column B. A case was considered to have a probable explanation if the organism was a well-recognized cause of the syndrome and there was one test from column B, or if the organism was not a well-recognized cause of the syndrome and there was one test from column A or 2 from column B. A case was considered to have a possible explanation if the organism was not a well-recognized cause of the syndrome and there was one test from column B, or if there was one test from column C, regardless of whether organism is known to cause the syndrome. ^b^"Involved” refers to the presence of typical pathology. ^c^IF = immunofluorescence, IHC = immunohistochemistry, IEM = immunoelectronmicroscopy, ISH = in situ hybridization, PCR = polymerase chain reaction, Ig = immunoglobulin, EM = electron microscopy, CSF = cerebrospinal fluid. ^d^for example, bronchoalveolar lavage in respiratory syndrome. ^e^EM is often nonspecific and may not permit reliable microbial identification without further characterization (e.g., IEM). ^f^Specific or broad range PCR/reverse transcriptase-PCR; product must be characterized beyond size determination (e.g., sequencing, single-strand conformation polymorphism, restriction fragment-length polymorphism, or probe hybridization).

## Statistical Analysis

Analysis was performed by SAS 6.12 (SAS Institute, Cary, NC). Denominators for the population under surveillance, obtained from the 1992 intercensus (*14*), included all persons in the age groups under surveillance at the various sites; denominators including only previously healthy persons are not available, and no attempt was made to estimate this fraction. Data from the surveillance population were standardized to the U.S. population by race and age to project the number of cases occurring nationally. The chi-square test was used to compare the distribution of characteristics between explained and unexplained cases. A p value ≤0.05 was considered statistically significant.

## Results

### Epidemiology

From May 1, 1995, to December 31, 1998, 525 possible cases were reported to UNEX personnel; 388 of these reports were excluded. The three most common reasons for exclusion were the presence of a preexisting medical condition (33%), residence outside the surveillance area (17%), and cause identified by local health-care providers on further testing (26%). Among cases excluded for the last reason, 72% had an infectious cause identified.

A total of 137 cases met the case definition, for a minimum overall annual rate of 0.5 per 100,000 population. After data were adjusted for age and race, this rate translates into 920 cases in the United States each year. The overall annual incidence rates remained stable over time, but varied among the different sites from 0.3 to 2.3 per 100,000 per year. The highest rate was in Connecticut, where active surveillance was conducted in a well-defined population of approximately 500,000 persons. Forty-one (30%) of the case-patients died, of whom 30 (73%) had autopsies performed, reflecting a rate much higher than the national autopsy rate of <11% (*15*). Cases were reported a median of 6 days from time of admission to the hospital (0 to 289 days).

**Figure 1:**
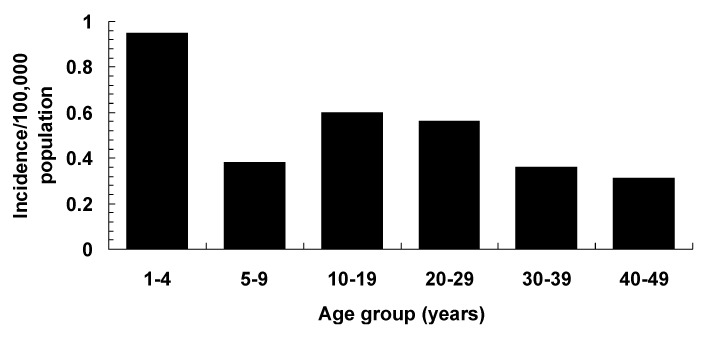
The incidence of cases by age group, 1995-1998, the Surveillance for Unexplained Deaths and Critical Illnesses Due to Possibly Infectious Causes Project (UNEX).

The median age of case-patients was 20 years; 20 (15%) were 1 to 4 years of age, 53% were female, and 82% were white. The incidence rates varied by age group (Figure 1) but did not differ by sex and race. No differences were observed in the seasonal distribution of cases, nor was there clustering of cases by time or place. As for exposures, 54% of all cases were reported to have pets, which is similar to national rates of pet ownership: 54% to 64% (American Veterinary Medical Association U.S. Pet Ownership and Demographics Sourcebook); 8% had traveled outside the United States in the year before hospitalization, and 4% had received transfusions at least once in their lifetime.

### Clinical Features

Table 2 summarizes the distribution of cases and the proportion explained by syndrome, as well as the syndrome-specific case-death ratios. The largest proportion of cases presented as a neurologic syndrome, followed closely by respiratory syndrome. The highest syndrome-specific case-death ratio was seen among cases with cardiac syndrome (46%) and the lowest among cases with neurologic syndrome (18%). An example of a case is described in Appendix III.

**Table 2:** Distribution of unexplained deaths and critical illness cases by syndrome, with proportion explained

Syndrome	No. (%)	No. of deaths (%)	No. of explained /cases with specimens (%)
Neurologic	39 (29)	7 (18)	15/37 (41)
Respiratory	36 (26)	11 (31)	13/33 (39)
Cardiac	28 (20)	13 (46)	3/22 (14)
Multisystem	18 (13)	4 (22)	3/15 (20)
Hepatic	9 (7)	4 (44)	0/8 (0)
Other	7 (5)	2 (29)	0/7 (0)
All cases	137	41 (30)	34/122 (28)

### Surveillance Audit

Table 3 summarizes the results of the surveillance audits. The site-specific sensitivity (S_D_) of our prospective surveillance for detecting unexplained deaths ranged from 38% in California to 100% in both Connecticut and Minnesota. Retrospective death record review identified 25% to 100% of all deaths detected through surveillance. Cases detected through surveillance but not by death record review were missed by the latter because the death certificates did not have the specified ICD-9 codes. The review of hospital discharge data focused on one tertiary-care referral hospital under surveillance in California and the one in Oregon, but included the entire surveillance area in Connecticut (*16*). Of potential cases identified by the selected ICD-9 codes, 90% to 96% were excluded, indicating the lack of specificity of these codes. The sensitivity of our prospective surveillance to detect only critical illnesses due to potentially infectious causes (S_C_) was 13% to 73%. Retrospectively, the hospital discharge review was able to identify 41% to 81% of all cases detected prospectively through our surveillance.

**Table 3:** Sensitivity of methods to identify cases of unexplained deaths and critical illnesses of possible infectious etiology, including the prospective surveillance conducted during this project and retrospective record reviews

	California	Oregon	Connecticut	Minnesota^1^
Sensitivity of prospective surveillance for unexplained deaths (%)	38	72	100	100
Proportion of all unexplained deaths identified retrospectively through death record review (%)	63	100	25	83
Sensitivity of prospective surveillance for critical illnesses(%)	25	13	73	
Proportion of critical illnesses identified retrospectively by hospital discharge data review (%)	75	81	41	-

^1^Minnesota did not review hospital discharge records.

### Search for Etiologic Agents

**Figure 2:**
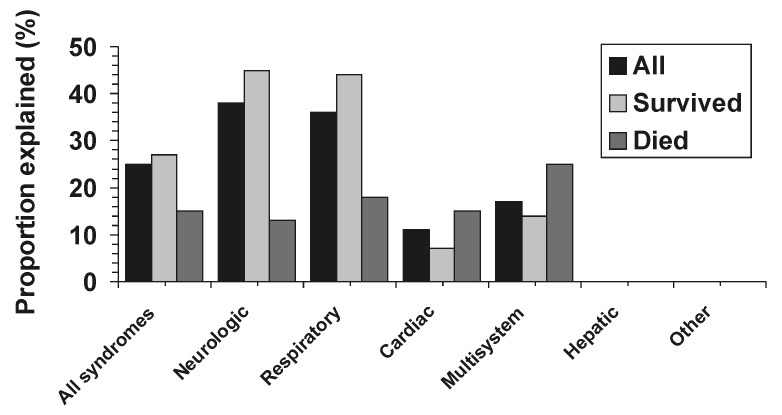
The explained proportions of cases by syndrome and survival status, 1995-1998, Surveillance for Unexplained Deaths and Critical Illnesses Due to Possibly Infectious Causes (UNEX).

Of the 137 UNEX case-patients, 122 had specimens available for testing; 10 of these had tissue specimens only. Of the 122 cases, 34 (28%) could be attributed to a specific infectious agent; these agents were classified as definite or probable causes of the illness, based on our criteria (Table 1). Specific infectious causes and the laboratory methods used for diagnosis are listed in Table 4. Table 5 lists additional infectious causes for possible cases that did not meet our criteria for definite or probable causation. All the infectious agents identified in this study were previously recognized bacterial and viral pathogens. One patient, admitted because of syncope, was found to have a complete heart block and had evidence of simultaneous infection with *Borrelia burgdorferi* and *Ehrlichia chaffeensis,* which has been previously reported (*17*). A number of cases met the clinical definition for various infectious diseases syndromes, including toxic shock syndrome (five cases), but did not meet our definition for an explained case. In addition, four cases had evidence of polyclonal serologic response to multiple infectious agents and therefore could not be attributed to a specific etiology. The proportion of explained cases was largest among those with neurologic syndromes, followed by those with respiratory syndromes; it was higher among surviving patients (29%) than among patients who died (15%), although this difference was not statistically significant (p=0.2) (Figure 2). Explained cases were similar to unexplained cases in terms of patient age, sex, and race, but were reported sooner after admission than unexplained cases (median 4 vs. 7.5 days, respectively; p=0.1). The proportion of explained cases during 1998 (7 [17%] of 41), when laboratory testing protocols were used routinely for first-round testing, did not differ significantly from the same proportion for cases enrolled during 1995-1997 (27 [28%] of 96) when no such protocol was used (p>0.05).

**Table 4:** Infectious disease causes for explained cases, UNEX,1995-1998, California, Oregon, Connecticut, and Minnesota (n=34)

Syndrome	Etiology (n)	Tests (n)
Neurologic (n=15)	*Neisseria meningitidis* (4)	16S rDNA PCR (2), PCR (1), EIA IgM (1)^a^
	*Bartonella henselae* (1)	PCR, IFA IgG
	*Bartonella* spp. (2)	IFA IgG
	*Chlamydia pneumoniae* (1)	MIF IgG
	*Mycoplasma pneumoniae* (1)	EIA IgM/IgG
	Cytomegalovirus (1)	EIA & IFA IgG
	Coxackie B (1)	EIA IgM, viral culture
	Enterovirus (1)	EIA IgM
	Epstein-Barr virus^b^ (1)	IFA IgG (VCA and EA)
	Human herpes virus 6 (1)	IFA and EIA (IgM and IgG)
	Mumps virus (1)	IFA IgM, IFA and EIA IgG
Respiratory (n=13)	*Chlamydia pneumoniae* (2)	MIF IgG (2), IFA IgM
	*Mycoplasma pneumoniae* (4)	PCR (blood), EIA IgM/IgG
	*Streptococcus pneumoniae* (2)	16S rDNA PCR (pleural fluid)
	*Legionella* spp. (1)	PCR (from lung)
	Adenovirus (1)	EIA and IFA IgG
	*Influenza B virus* (1)	EIA and IFA IgG
	*Influenza A virus* (1)	EIA and IFA IgM, EIA (IgG)
	Parainflueza virus types 1 and 3 (1)	EIA and IFA IgG
Cardiac (n=3)	*Borrelia burgdorferi/Ehrlichia chaffeensis* (1)	EIA*/* IFA flagella IgG, Western blot (IgG, IgM)
	*Enterovirus* (1)	EIA IgM
	*Legionella* spp. (1)	PCR (heart)
Multisystem (n=3)	*Neisseria meningitidis* (1)	PCR (CSF)
	Adenovirus (1)	PCR (blood)
	*Enterovirus* (1)	IgM EIA

^a^EIA = enzyme immunosorbent assay, IFA = indirect immunofluorescent assay, IG = immunoglobulin, MIF = microimmunofluorescence, PCR = polymerase chain reaction. ^a^See Appendix III for a detailed description of this case.

**Table 5:** Infectious causes for “possibly” explained cases, UNEX, 1995-98, California, Oregon, Connecticut, and Minnesota (n=34)

Syndrome	Etiology (n)	Tests (n)
Neurologic	*Mycoplasma pneumoniae*	Remel EIA (IgM/IgG)^a^
	*Influenza B virus* (FLUBV)	Nasopharyngeal culture
	Varicella-zoster virus (reactivation)	EIA/IFA IgG
Respiratory	*Enterovirus*	EIA IgM
Cardiac	*Chlamydia pneumoniae*	MIF IgG
	Adenovirus	EIA IgM
	FLUBV	IFA IgM
Other^b^	Enterovirus (2)	IgM EIA

^a^EIA = enzyme immunosorbent assay, IFA = indirect immunofluorescent assay, IG = immunoglobulin, MIF = microimmunofluorescence, PCR = polymerase chain reaction. ^b^Other syndromes included one case with thrombotic thrombocytopenic purpura and one with hemolytic uremic syndrome.

Clinical specimens from each enrolled patient underwent an average of 28 laboratory tests (up to 103 tests). The mean number of tests performed did not differ substantially for explained and unexplained cases (30 vs. 27, respectively). None of the cases with only histologic specimens available had an infectious cause identified. Of the 34 explained cases, 23 (68%) were explained by using serologic tests, 7 (21%) by specific primer PCR, and 4 (12%) by 16S rDNA PCR. Among the 122 cases with specimens, serologic testing provided the highest yield in identifying infectious causes (23 [22%] of 104), followed by specific primer PCR (7 [10%] of 70) and 16S rDNA PCR (4 [8%] of 48). An infectious etiology was more likely to be identified in cases with paired serum specimens (14 [23%] of 62) than in those with single serum specimens (2 [5%] of 42) (p=0.05).

## Discussion

This study is the first to measure the population burden of unexplained deaths and critical illness from possibly infectious causes in the United States. To our knowledge, this is the first public health attempt to describe the features of this problem, in spite of its clinical complexities. This project established the infrastructure needed to detect UNEX cases, attempt to identify their etiology, and ultimately identify new infectious agents. However, since this project was a pilot study, it was difficult to standardize many of its aspects. Many lessons were learned during this project, whether related to the best surveillance methods to use or the laboratory testing process. In addition, data obtained in the first 3.5 years of this project suggest that UNEX occur in previously healthy persons at rates similar to those of other conditions of clear public health concern and priority (*18*). Of obvious concern is also the large proportion of these deaths and severe illnesses that remains unexplained after extensive laboratory testing. Our findings highlight the substantial limitations of available diagnostic tests for infectious diseases and the need for improved tests and novel approaches to identify infectious disease agents.

Our surveillance estimated the burden of disease only among previously healthy persons 1 to 49 years of age. Since a different age cut-off was used in Oregon, the final rates of disease were adjusted for age and race. The lower age limit was chosen to avoid confusion with congenital problems seen in infants but to include most children in day care, where infectious diseases are common and new infectious diseases might spread rapidly. The upper age limit was intended to exclude an expected increased proportion of unexplained deaths due to noninfectious causes among persons ≥50 years of age. Although immunocompromised patients are more susceptible to a variety of infectious diseases, available resources and a concern that the clinical relevance of novel microbial findings would be more difficult to interpret in immunocompromised persons compelled us to focus on previously healthy persons. In addition, many of the new infectious diseases first identified in these persons have subsequently been found to affect persons with normal immune systems (*19*,*20*).

The surveillance methods adopted during this project were customized to meet the objectives of this study, taking into consideration the limitations of local resources; therefore UNEX cannot be easily compared with other classical surveillance systems. The different methods of surveillance used at the four sites allowed us, through the surveillance audits and validation, to determine how these differences affected case-finding. For example, investigators in Connecticut were able to detect most UNEX cases largely because they conducted more active surveillance in a smaller population base; in this site, surveillance focused on all seven hospitals in New Haven County. At the academic tertiary-care hospital, EIP staff reviewed ICU admission logs and communicated with clinicians daily. At the other six hospitals, a stimulated passive surveillance system was used in which physicians and infection control practitioners were given reminders several times per year. The active prospective method captured a greater proportion of total cases (86% of cases at the single hospital) then did the passive methods (50% of total cases at the six remaining hospitals).

If this surveillance is to be expanded, different methods may be chosen, depending on availability of resources and overall objectives. Less resource-intensive passive surveillance may be used if the goal is to monitor trends in disease occurrence. For example, although analyzing all death certificates for UNEX cases would be prohibitively time-consuming, electronically searching only the certificates in which the manner of death was recorded as natural, undetermined, or pending investigation could substantially decrease the workload. Under a passive system, maintaining good communication between study staff and clinical staff (clinicians, pathologists, and infection control practitioners) is critical and aided by the provision of diagnostic testing not locally available (such as serologic testing for hantavirus or toxin testing for botulism) and timely feedback of study results. Such collaboration may be critical to early diagnosis of diseases that produce characteristic clinical syndromes (e.g., potential bioterrorist agents such as botulism) or that are not readily confirmed by clinical laboratories.

Before initiating this project, we had reviewed multiple cause-of-death data for the United States to estimate the number of unexplained deaths from possibly infectious causes at these EIP sites (*3*). In 1992, the rate of unexplained deaths among healthy persons 1 to 49 years of age was 8.9 per 100,000 population. The discrepancy between this rate and that found in our study (0.5 per 100,000) is likely due to the low specificity of ICD-9 codes in excluding persons with previous health problems, as well as the problems related to retrospective analysis in general. For at least two reasons, we expect that the incidence of UNEX found in this study represents only a minimal estimate of the true burden of this problem. First, the denominator in our calculations included all persons in our designated groups, since we chose not to estimate the fraction of previously healthy persons in the surveillance populations at the four sites. Second, the differences in incidence rates between the four surveillance sites and results of the surveillance audits support the assumption that the overall rate detected was a minimal estimate of overall disease.

An important unresolved issue from our study is the large proportion of cases that remained unexplained, even after extensive laboratory testing. Although a standardized protocol for testing was used only during 1998, the proportion of explained cases before and after this protocol was used did not differ substantially. Some illnesses may have noninfectious causes, especially given the lack of specificity in our clinical criteria for case inclusion and in the features of infection in general. In cardiac syndromes, for example, myocarditis and myocardial infarction can have very similar presentations. Some cases may have been caused by microbial products such as toxins without the presence of the organism or substantial amounts of its nucleic acids. Laboratory methods for screening and detection of toxins remain inadequate. For some patients, specimens were not available from the primary site of disease, were severely limited in quantity, or were only available from late in the course of the disease; in many cases, multiple serum specimens were not available, autopsies were incomplete, and tissue specimens were obtained only from unaffected organs. Finally, the breadth of our testing methods may not have been adequate. Since broad-range PCR methods were applied only to bacteria and a limited range of viruses, many other potential agents may have been missed. Our approach to the detection of viral pathogens relied more heavily on serologic and immunohistochemical techniques, in part because of the difficulty in designing a comprehensive set of consensus PCR primers for all known viral families (*21*). In our study, viral testing was also constrained by limited experience with certain IgM assays. The development, testing, and application of comprehensive broad-range viral and fungal consensus primers for use in PCR assays may be helpful. Through this project, we created a population-based bank of clinical specimens that may prove valuable in the search for newly recognized etiologic agents, the development of diagnostic tests, and the standardization of nucleic acid-based techniques for identifying previously unknown etiologic agents.

This project represents an attempt to build capacity for early detection and response to emerging infectious diseases threats in the United States and elsewhere. The usefulness of this surveillance system for UNEX was recently illustrated during an outbreak of *West Nile virus* encephalitis in the northeastern United States (*22*) and an outbreak of unexplained illness among injecting drug users in Scotland and Ireland (*23*); initial reports of illness from both these investigations were received through the UNEX surveillance project, and initial testing was conducted through the infrastructure developed for this project. Future surveillance for UNEX may benefit from simplified case-finding methods, improved specimen quality, and more focused syndrome-specific surveillance. Once validated, surveillance methods may be adopted by the broader public health community. Such surveillance approaches will strengthen the collaboration between clinicians, laboratorians, and public health professionals, leading to improved detection of unexplained deaths and critical illnesses, including possible bioterrorism events, and better monitoring of emerging infectious diseases.

## Supplementary Material

Appendix I Case Definition, Surveillance for Unexplained Deaths and Critical Illnesses Due to Possibly Infectious Causes, United States, 1995-1998

Appendix II Standardized Syndrome-specific Laboratory Testing Protocols

Appendix III An Example of a Clinical Case Surveillance for Unexplained Deaths and Critical Illnesses Due to Possibly Infectious Causes, United States, 1995-1998

## Footnotes

1 Divya Agrawal, William Bower, Richard Danila, Linda Duke, Mark Eberhard, Marc Fischer, Jeannette Guarner, Connie Heye, James Johnson, Rima Khabbaz, Fred Lopez, Ruth Lynfield, James Meek, Christopher Paddock, Arthur Reingold, Robert Ryder, Susan Smith, Deborah F. Talkington, Michael Virata, and Duc Vugia.

2 Oregon used a different age cut-off because of limited resources.

3 IHC was available at CDC for the following pathogens: *Acanthamoeba culbertzoni*; adenovirus; *Bacillus anthracis; Balamuthia* spp.; *Bartonella henselae, Bartonella quintana; Brucella* spp.; *Chlamydia* spp.; *Coccidiodes* spp; *Coxiella burnetii*; *Crimean-Congo hemorrhagic fever virus*; *Cryptococcus* spp.; *Cytomegalovirus*; *Dengue virus*; *Eastern equine encephalitis virus*; Ebola virus; *Ehrlichia chaffeensis*; *Enterovirus* (Pan-enterovirus); human enterovirus 71; *Flavivirus*; Japanese encephalitis serocomplex group (*West Nile virus*, *St. Louis encephalitis virus* [SLEV], *Japanese encephalitis virus*); *Francisella tularensis*; Group A streptococci; *Guanarito virus* (Venezuelan hemorrhagic fever virus); *Hantavirus*; *Helicobacter pylori*; Hendra virus; herpes simplex viruses 1 and 2; *Histoplasma* spp.; human granulocytic ehrlichiosis; *Human herpesvirus 6*; HIV-1; HIV-2; *B19 virus* (B19V); *Influenza A virus* (FLUA); *Influenza B virus* (FLUB); *Junin virus* (Argentine hemorrhagic fever); La Crosse virus; *Lassa virus*; *Legionella pneumophila* serogroups 1, 5, 6; *Leptospira* spp.; *Listeria monocytogenes*; *Lymphocytic choriomeningitis virus* (LCMV); *Machupo virus* (Bolivian hemorrhagic fever); *Marburg virus*; *measles (Edmonston) virus* (MeV); *Mycobacterium* spp.; *Mycoplasma pneumoniae*; *Naegleria fowleri; Neisseria meningitidis* C; Nipah virus; *Human parainfluenza virus types 1 and 3* (HPIV 1,3); *Rabies virus* (RABV); *Human respiratory syncytial virus* (HRSV); *Rickettsia* spp. *Orientia* group; *Rickettsia* spp. spotted fever group; *Rickettsia* spp. Typhus group; *Rift Valley fever virus*; *Rotavirus*; *Streptococcus pneumoniae*; *Toxoplasma gondii; Treponema pallidum; Trypanosoma cruzi*; varicella-zoster virus (VZV); *Venezuelan equine encephalitis virus*; *Western equine encephalomyelitis virus* (WEEV); *Yellow fever virus*; and *Yersinia pestis.*

4 The following viral tests were used at the CDHS Viral and Rickettsial Diseases Laboratory: IgG was detected by both EIA and IFA for adenovirus, HHV-6, HHV-8, herpes simplex virus, FLUAV, FLUBV, MeV, *Mumps virus* (MuV), HPIV-1-4, HRSV, *Rubella virus* (RUBV), VZV, SLEV, and WEEV. IgG was detected by EIA only for *Hantavirus* (*Sin Nombre virus* [SNV]) and B-19. IgG was detected by IFA only for Epstein-Barr virus (viral capsid antigen), LCV, and RABV. IgM was detected by both EIA and IFA for HHV-6, herpes simplex virus, MeV, MuV, HPIV-1-4, HRSV, RUBV, and VZV. IgM was detected by EIA only for enterovirus, hantavirus (SNV), and B19V. IgM was detected by IFA only for Epstein-Barr virus. PCR tests performed were Herpesvirus consensus PCR (6); enterovirus PCR, modified from (7) [Antisense primer (1R): 5’-ATT GTC ACC ATA AGC AGC CA, sense primer (1L): 5’-CCT CCG GCC CCT GAA TGC GGC TAA T]; and adenovirus PCR (8).
