# Supplementary material for: Surveillance for Unexplained Deaths and Critical Illnesses
Source: Emerg Infect Dis. 2002 Feb;8(2):145–53. doi: 10.3201/eid0802.010165 (PMC2732455; doi:10.3201/eid0802.010165)
Supplement: Appendix I — Case Definition, Surveillance for Unexplained Deaths and Critical Illnesses Due to Possibly Infectious Causes, United States, 1995-1998 [file 01-0165_app-s1.pdf]

Publisher: CDC; Journal: Emerging Infectious Diseases
Article Type: Research; Volume: 08; Issue: 02; Year: 2002; Article ID: 01-0165
DOI: 10.321/eid0802.010165; TOC Head: Research

Appendix I

Case Definition, Surveillance for Unexplained Deaths and Critical Illnesses Due to Possibly Infectious Causes, United States, 1995-1998

Previously Healthy

Patients are considered previously healthy who had no known preexisting chronic medical condition before the onset of the illness resulting in hospitalization or death, including malignancy; HIV infection; chronic cardiac; pulmonary, renal, hepatic, or rheumatologic disease; or diabetes mellitus. These patients have no history of immunosuppressive therapy, trauma thought to be related to illness, evidence of toxic ingestion or exposure, or nosocomial infection.

Reasons for Exclusion

1. A history of a malignancy other than nonmelanoma skin cancer
2. HIV infection identified during hospitalization, previously or after discharge
3. History of physician-confirmed myocardial infarction, angina with known coronary artery disease, or congestive heart failure
4. Any history of hospital admission for asthma or other pulmonary diseases except for uncomplicated pneumonia
5. History of dialysis or chronically elevated blood urea nitrogen and creatinine

6. Biopsy-proven liver disease of any kind or chronic coagulopathy or chronic *Hepatitis B* or *C virus* infection as a result of hepatic insufficiency
7. Physician-confirmed rheumatologic conditions requiring chronic or intermittent medical therapy with oral steroids or other immunosuppressive drugs
8. Any known physician-confirmed diabetes mellitus previously or during hospitalization
9. Development of hallmarks of infection >48 hours after hospital admission
10. Any mention of a history of excessive alcohol use, alcohol abuse, or alcoholism is a reason for exclusion.(e.g., delirium tremens, withdrawal seizures, alcoholic neuropathy, persistent liver function test abnormalities, gastrointestinal bleeding, coagulopathy, or hypoalbuminemia).
11. Any mention of injecting drug use
12. Any history of neurologic disease, including seizures,
13. Obesity, defined as body mass index ≥30 or “obese” noted in medical chart
14. Physician-confirmed diagnosis of anorexia

Not Reasons for Exclusion

1. Hypertension or a history of hypertension
2. Any history of inhaler use
3. Pyelonephritis or nephrolithiasis or a history of either of these conditions in the absence of a chronically elevated blood urea nitrogen and creatinine
4. History of hepatitis
5. Pregnancy
