# Supplementary material for: Surveillance for Unexplained Deaths and Critical Illnesses
Source: Emerg Infect Dis. 2002 Feb;8(2):145–53. doi: 10.3201/eid0802.010165 (PMC2732455; doi:10.3201/eid0802.010165)
Supplement: Appendix II — Standardized Syndrome-specific Laboratory Testing Protocols [file 01-0165_app-s2.pdf]

Algorithm for Meningo-Encephalitis

Specimens (brain, meninges, spinal cord, nerve) will be sent to Centers for Disease Control and Prevention (CDC) for testing by monoclonal antibody, hybridization, electron microscopy, or polymerase chain reaction (PCR). Before any testing, 250 µL (or other amount) from each specimen will be stored in a serobank. First-round tests will be performed on all specimens. Culture for bacterial and viral organisms are to be performed at primary site.

Date of symptom onset __/__/__
Date of hospital admission __/__/__

Available specimens (with dates)
- Serum
- Blood
- Cerebrospinal fluid (CSF)
- Blood culture
- Pleural fluid
- Pericardial fluid
- Peritoneal fluid
- Other

First-Round Testing

Viral Panel

Viral PCR preferable if acute-phase samples available (CSF, serum, whole blood, saliva [for rabies ≥1.0 mL])
- Adenovirus (100 µL serum, CSF)
- Cytomegalovirus
- *Enterovirus (coxsackie A and B, Echo, etc.)*
- Epstein-Barr Virus
- Herpes simplex virus (HSV) (150 µL serum, CSF)
- *Rabies virus* (RABV) (saliva)
- *Varicella-zoster virus (VZV)*
- *Human herpesvirus (HHV-6)* (for children <3 years of age)

Arboviruses
- California encephalitis group
- *Colorado tick fever virus* (CTFV)
- *Eastern equine encephalomyelitis virus* (EEEV)
- *St. Louis encephalitis virus* (SLEV)
- *Venezuelan equine encephalomyelitis virus* (VEEV)
- *Western equine encephalomyelitis virus* (WEEV)

Serologic testing on CSF and serum--(0.25 mL)
- Adenovirus immunoglobulin (Ig) M (enzyme immunoassay [EIA]-2.5 µL serum, immunofluorescent assay [IFA]-30 µL serum)

Adenovirus IgG (EIA-2.5 µL serum, IFA-50 µL serum)
Cytomegalovirus IgM (EIA-2.5 µL serum, IFA-30 µL serum)
Cytomegalovirus IgG (EIA-2.5 µL serum, IFA-50 µL serum)
Epstein-Barr virus IgM (IFA-30 µL serum)
Epstein-Barr virus IgG (IFA-50 µL serum)
Epstein-Barr nucleocapsid antigen (IFA-100 µL serum)
Enterovirus IgM *(Coxsackie, Echo)* (EIA 5 µL serum, 50 µL CSF)
HSV IgM (EIA-2.5 µL, IFA-30 µL serum)
HSV IgG (EIA-2.5 µL, IFA-50 µL serum)
Influenza A virus (FLUAV) IgM (EIA-2.5 µL serum, IFA-30 µL serum)
FLUAV IgG (EIA-2.5 µL serum, IFA-50 µL serum)
Influenza B virus (FLUBV) IgM (EIA-2.5 µL serum, IFA-30 µL serum)
FLUBV IgG (EIA-2.5 µL serum, IFA-50 µL serum)
Measles (Edmonston) virus (MeV) IgM (EIA-2.5 µL, IFA-30 µL serum)
MeV IgG (EIA-2.5 µL, IFA-50 µL serum)
Human parainfluenza virus (HPIV 1-4) IgM (complement fixation [CF]-serum, EIA-2.5 µL serum, IFA-30 µL serum)
HPIV 1-4 IgG (CF-serum, EIA-2.5 µL serum, IFA-50 µL serum)
RABV IgG (IIF-50 µL serum, CSF; NT-50 µL serum, CSF)
VZV IgM (EIA-2.5 µL, IFA-30 µL serum)
VZV IgG (EIA-2.5 µL, IFA-50 µL serum)

Arboviruses
California group IgG (IFA capture-50 µL serum, CSF)
EEEV IgM (enzyme-linked immunosorbent assay [ELISA] capture-2.5 µL serum, CSF)
EEEV IgG (ELISA capture-2.5 µL serum, CSF)
Powassan virus (POWV) IgM (ELISA capture-2.5 µL serum, CSF)
POWV IgG (ELISA capture-2.5µL serum, CSF)
SLEV IgM (ELISA capture-2.5 µL serum, CSF)
SLEV IgG (ELISA capture-2.5 µL serum, CSF)
VEEV IgM (ELISA capture-2.5 µL serum, CSF)
VEEV IgG (ELISA capture-2.5 µL serum, CSF)
WEEV IgM (ELISA capture-2.5 µL serum, CSF)
WEEV IgG (ELISA capture-2.5 µL serum, CSF)
HHV-6 IgM (EIA-2.5 µL serum, IFA-30 µL serum) (for children < 3 years of age)
HHV-6 IgG (IFA-50 µL serum)

Bacterial panel
Criteria: CSF PMNs >400, total protein >100, glucose <40, sudden death, prior antibiotic use
CSF--(0.5 mL)
Borrelia burgdorferi IgM (ELISA-5 µL CSF)
B. burgdorferi IgG (ELISA-5 µL CSF)
Haemophilus influenzae antigen (Ag) (LA-50 µL CSF)
Mycoplasma pneumoniae IgM/IgG (Remel EIA-50 µL serum)
Neisseria meningitidis PCR (100 µL)

N. meningitidis Ag (LA-A,C,Y,W-135-250 µL CSF)
Streptococcus pneumoniae Ag (LA-50 µL CSF)
Syphilis (VDRL-CSF)

Serologic testing--(0.5 mL)
B. burgdorferi IgM (ELISA-5 µL serum)
B. burgdorferi IgG (ELISA-5 µL serum)
H. influenzae Ag (LA 50 µL)
H. influenzae IgM (ELISA-30 µL serum)
H. influenzae IgG (ELISA-30 µL serum)
Leptospira spp. (IgM/IgG MAT- 60 µL,IgM ELISA-5 µL)
M. pneumoniae IgM/IgG (Remel EIA-50 µL serum)
M. pneumoniae IgG (Zeus EIA-50 µL serum)
N. meningitidis Ag (LA-A,C,Y,W-135-250 µL serum)
N. meningitidis (ELISA-100 µL serum)
N. meningitidis A IgM
N. meningitidis A IgG
N. meningitidis B IgM
N. meningitidis B IgG
N. meningitidis C IgM
N. meningitidis C IgG
N. meningitidis Y IgM
N. meningitidis Y IgG
N. meningitidis W-135 IgM
N. meningitidis W-135 IgG
Syphilis (RPR-serum, FTA-ABS)

Serologic testing for *Bartonella* spp.
Bartonella elizabethae IgM (IFA-30 µL blood)
B. elizabethae IgG (IFA-50 µL blood)
B. henselae IgM (IFA-30 µL blood)
B. henselae IgG (IFA-50 µL blood)
B. quintana IgM (IFA-serum)
B. quintana IgG (IFA-serum)

Fungal panel (0.5 mL)
Cryptococcis neoformans Ag (LA-350 µL urine, serum, CSF)
Endemic Fungal Panel (CF-75 µL- serum, CSF; ID-100 µL serum, CSF)
Coccidioides immitis (CF, ID, ELISA-serum, CSF)
Blastomyces dermatididis (CF, ID, ELISA-serum, CSF)
Histoplasma capsulatum (CF, ID-serum , CSF)
H. capsulatum Ag (RIA-urine, serum, CSF; molecular probe-serum)
Paracoccidioides brasiliensis (CF, ID-serum, CSF)

Rash: (0.5 mL)

Erythema
Toxic Shock Syndrome Toxin-1
Maculopapular
Ehrlichia chaffeensis IgM (IFA-30 µL serum)
E. chaffeensis IgG (IFA-50 µL serum)
E. phagocytophilia (HGE) IgM (IFA-30 µL serum)
E. phagocytophilia (HGE) IgG (IFA-50 µL serum)
Rickettsia rickettsii IgM (IFA-30 µL serum)
R. rickettsii IgG (IFA-50 µL serum)
R. typhi IgM (IFA-30 µL serum)
R. typhi IgG (IFA-50 µL serum)
Rubella virus (RUBV) IgM (EIA-2.5 µL, IFA-30 µL serum)
RUBV IgG (EIA-2.5 µL, IFA-50 µL serum)
Vesiculonodular
Variola IgM (EIA-2.5 µL, IFA-30 µL serum)
Variola IgG (EIA-2.5 µL, IFA-50 µL serum)

Eosinophilia >8% or eosinophils in CSF:
Paragonimus westermanii (IB-serum)
Schistosoma spp. IgM/IgG (screening EIA-serum, speciate IB-serum)
Strongyloides stercoralis (serum)
Taenia spp. (cysticercosis) (IB-serum, CSF)
Toxocara spp. IgM/IgG (EIA,-serum)
Trichinella spiralis IgM/IgG (EIA, bentonite flocculation-serum)

Hemorrhage: (0.5 mL)
with history of travel to Africa or South America
Hemorrhagic fever panel (*Crimean Congo hemorrhagic fever virus* [C-CHFV], *Lassa virus* [LASV], *Machupo virus* [MACV], *Junin virus* [JUNV], *Rift Valley fever virus* [RVFV], *Marburg virus* [MBGV], Ebola virus [EBOV], *Omsk hemorrhagic fever virus* [OHFV])
Hemorrhagic fever Ag (ELISA 250 µL serum)
Hemorrhagic fever IgM (ELISA 100 µL serum)
Hemorrhagic fever IgG (ELISA 100 µL serum)

High-Priority Second Round (if not previously performed)

Bacterial PCR (CSF and serum)--(0.5 mL)
Mycobacterium tuberculosis
Neisseria meningitidis PCR (100 µL)
16S universal probe (serum, whole blood, CSF)-- (100 µL)

Bacterial serologic testing
Bacterial tests--(1.0 mL)
Borrelia recurrentis IgM (ELISA-5 µL serum)
B. recurrentis IgG (ELISA-5 µL serum)

Brucella spp. (MAT-serum 20 µL)
Chlamydia pneumoniae IgM (MIF-10 µL serum, EIA-2.5 µL serum, IFA-30 µL serum)
C. pneumoniae IgG (MIF-10 µL serum, EIA-2.5 µL serum, IFA-50 µL serum)
Chlamydia psittaci IgM (MIF-10 µL serum, EIA-2.5 µL serum, IFA-30 µL serum)
C. psittaci IgG (MIF-10 µL serum, EIA-2.5 µL serum, IFA-50 µL serum)
Legionella pneumophila IgM (IFA-25 µL serum, ELISA-10 µL serum)
L. pneumophila IgG (IFA-25 µL serum, ELISA-10 µL serum)
L. pneumophila Ag (RIA-100 µL urine)
Listeria IgM (ELISA-30 µL serum)
Listeria IgG (ELISA-30 µL serum)

Rickettsial serologic testing--(0.5 mL)
Coxiella burnetti IgM (IFA-5 µL serum)
C. burnetti IgG (IFA-50 µL serum)
Ehrlichia canis IgM (IFA-30 µL serum)
E. canis IgG (IFA-50 µL serum)
E. chaffeensis IgM (IFA-30 µL serum)
E. chaffeensis IgG (IFA-50 µL serum)
E. phagocytophilia (HGE) IgM (IFA-30 µL serum)
E. phagocytophilia (HGE) IgG (IFA-50 µL serum)
Rickettsia rickettsii IgM (IFA-30 µL serum)
R. rickettsii IgG (IFA-50 µL serum)
R. typhi IgM (IFA-30 µL serum)
R. typhi IgG (IFA-50 µL serum)

Viral PCR--
Cache Valley virus (CVV)
Jamestown Canyon virus (JCV)
Murray Valley encephalitis virus (MVEV)
Powassan virus (POWV)
Snowshoe hare virus (SSHV)
Tickborne complex

Viral serologic testing (serum)--(1.0 mL)
Lymphocytic choriomeningitis virus (LCM) Ag (EIA-50 µL serum)
LCM IgM (IF-serum)
LCM IgG (IF-serum)
MeV IgM (EIA-2.5 µL, IFA-30 µL serum)
MeV IgG (EIA-2.5 µL, IFA-50 µL serum)
Mumps virus (MuV) IgM (EIA-2.5 µL, IFA-30 µL serum)
MuV IgG (EIA-2.5 µL, IFA-50 µL serum)
RUBV IgM (EIA-2.5 µL, IFA-30 µL serum)
RUBV IgG (EIA-2.5 µL, IFA-50 µL serum)

Parasitology serologic testing--(0.5 mL)
Babesia microti (IFA-serum)
Babesia WA-1 (IFA-serum)
Malaria IgG (IFA-serum)
Strongyloides stercoralis (serum)

Other tests to consider
Viral PCR--
HTLV-I
with travel to Africa
Rift Valley fever virus (RVFV)
West Nile virus (WNV)
with travel to Asia
Chikungunya virus (CHIV)
Hendra virus (HeV)
Japanese encephalitis virus (JEV)
Kyanasur Forest disease virus (KFDV)

Viral serologic testing--(0.5 mL)
CVV Ag (ELISA 50 µL serum)
CVV IgM (ELISA 5 µL serum)
CVV IgG (ELISA 5 µL serum)
CHIV IgM (ELISA 5 µL serum)
CHIV IgG (ELISA 5 µL serum)
Dengue virus (DENV) (1-4) IgM (IF-serum)
DENV (1-4) IgG (IF-serum)
JCV IgM (ELISA 5 µL serum)
JCV IgG (ELISA 5 µL serum)
JEEV IgM (ELISA 5 µL serum)
JEEV IgG (ELISA 5 µL serum)
KFDV IgM (ELISA 5 µL serum)
KFDV IgG (ELISA 5 µL serum)
RVFV IgM (ELISA 5 µL serum)
RVFV IgG (ELISA 5 µL serum)
Tickborne complex IgM (ELISA 5 µL serum)
Tickborne complex IgG (ELISA 5 µL serum)
WNV IgM (ELISA 5 µL serum)
WNV IgG (ELISA 5 µL serum)
Yellow fever virus (YFV) Ag (ELISA 50 µL serum)
YFV IgM (ELISA 5 µL serum)
YFV IgG (ELISA 5 µL serum)

HTLV-I IgM (ELISA 5 µL serum)
HTLV-I IgG (ELISA 5 µL serum)
Hepatitis A virus (HAV) IgM (EIA-10 µL serum)

Hepatitis B virus (HBV) surface Ag (EIA-150 µL serum)
HBV surface Ab (EIA-200 µL serum)
HBV core IgM (EIA -10 µL serum)
HBV core total (EIA-100 µL serum)
HBV eAg (EIA-200 µL serum)
HBV eAb (EIA-100 µL serum)
Hepatitis C virus (HCV) IgM/IgG (EIA-30 µL serum with confirmation)
Hepatitis delta virus (HDV) IgM (EIA-10 µL serum)
HDV total (EIA-100 µL serum)
HDV Ag (EIA-50 µL serum)
Hepatitis E virus (HEV) IgM (EIA-50 µL serum)
HEV IgG (EIA-10 µL serum)

Bacterial tests--(1.0 mL)
Afipia felis IgM (IFA-30 µL blood)
A. felis IgG (IFA-50 µL blood)
Tropheryma whippelii IgM (IFA-30 µL blood)
T. whippelii IgG (IFA-50 µL blood)

Bartonella tests
B. quintana PCR (EDTA blood)
B. elizabethae PCR (EDTA blood)
B. henselae PCR (EDTA blood)
with travel to Latin America
B. bacilliformis PCR (EDTA blood)
B. bacilliformis IgM (IFA-30 µL blood)
B. bacilliformis IgG (IFA-50 µL blood)

Rickettsial serologies--(0.5 mL)
Rickettsia akari IgM (IFA-30 µL blood)
R. akari IgG (IFA-50 µL blood)
R. prowazekii IgM (IFA-30 µL blood)
R. prowazekii IgG (IFA-50 µL blood)
with travel to Africa
R. africae IgM (IFA-30 µL blood)
R. africae IgG (IFA-50 µL blood)
R. conorii IgM (IFA-30 µL blood)
R. conorii IgG (IFA-50 µL blood)
with travel to SE Asia, SW Pacific, Australia
Orientia tsutsumagushi IgM (IFA-30 µL blood)
O. tsutsumagushi IgG (IFA-50 µL blood)

Rickettsial PCR--(0.5 mL)
Ehrlichia chaffeensis
E. phagocytophilia (Human granulocytic ehrlichiosis)

O. tsutsumagushi
R. africae
R. akari
R. conorii
R. prowazekii
R. rickettsii
R. typhi

Parasitic serologic testing--(0.5 mL)
Paragonimus westermani (IB-serum)
Schistosoma spp. IgM/IgG (screening EIA-serum, speciate IB-serum)
Taenia spp. (cysticercosis) (IB-serum, CSF)
Trichinella spiralis IgM/IgG (EIA, bentonite flocculation-serum)
Toxocara spp. IgM/IgG (EIA,-serum)
