# Supplementary material for: Surveillance for Unexplained Deaths and Critical Illnesses
Source: Emerg Infect Dis. 2002 Feb;8(2):145–53. doi: 10.3201/eid0802.010165 (PMC2732455; doi:10.3201/eid0802.010165)
Supplement: Appendix III — An Example of a Clinical Case Surveillance for Unexplained Deaths and Critical Illnesses Due to Possibly Infectious Causes, United States, 1995-1998 [file 01-0165_app-s3.pdf]

Publisher: CDC; Journal: Emerging Infectious Diseases
Article Type: Research; Volume: 08; Issue: 02; Year: 2002; Article ID: 01-0165
DOI: 10.321/eid0802.010165; TOC Head: Research

Appendix III

An Example of a Clinical Case Surveillance for Unexplained Deaths and Critical Illnesses Due to Possibly Infectious Causes, United States, 1995-1998

A 22-month-old boy from Oregon was healthy except for previous bouts of otitis media, for which tympanostomy tubes had been placed. Three days before admission, in May 1997, tactile fever was noted, and one day before admission, the patient had decreased activity and rhinorrhea. On the day of admission, he vomited twice. In the emergency room, he had a temperature of 39.2°C, was irritable and lethargic, and had nuchal rigidity. A complete blood count showed a total leukocyte count (WBC) of 18,300 (69% segmented cells, 8% bands). Cerebrospinal fluid (CSF) analysis showed a WBC of 54 (25% segmented cells, 75% monocytes), protein 38 mg/dL, and glucose 70 mg/dL. The patient was hospitalized and initially treated with ceftriaxone. On the next day, he became less responsive, and abnormal posturing developed in the left upper and lower extremities. A computed tomography scan of the head (without and with contrast) was normal. He was transferred to a tertiary-care center, where an electroencephalogram showed moderate generalized slowing and recurrent right hemispheric electrographic seizures. A magnetic resonnance imaging scan done on the same day showed a diffusely increased white matter signal consistent with viral encephalitis or acute disseminated encephalomyelitis. The patient received acyclovir for 3 days. His responsiveness and clinical condition gradually improved, and he was transferred to a rehabilitation service 17 days after admission. Initial work-up at the hospital revealed negative blood cultures and negative bacterial and viral cultures of the CSF. PCR for Epstein-Barr virus in the blood and CSF was negative, as was PCR for Herpes simplex virus in CSF.

The patient was enrolled in the UNEX project and evaluated. Specimens available for testing included acute- and convalescent-phase serum and CSF specimens. A variety of tests were conducted (see neurologic syndrome testing protocol in Appendix II available online at http://www.cdc.gov/ncidod/eid/vol8no2/pdf/01-0165-app2.pdf). Because the quantities of

specimens available were limited, testing was prioritized. First-round testing was negative for *Cytomegalovirus*, HHV-6, and arboviruses. However, testing for IgG antibodies (by IFA) for Epstein-Barr viral capsid antibodies showed a fourfold rise in titer between acute- and convalescent-phase serum specimens; testing for IgG antibodies (also by IFA) to Epstein-Barr early antigen revealed a fourfold decrease in titer between convalescent- and acute-phase serum specimens, indicating acute Epstein-Barr infection.
